# Why women have more autoimmune diseases than men: An evolutionary perspective

**DOI:** 10.1111/eva.13167

**Published:** 2020-12-01

**Authors:** Vanessa L. Kronzer, Stanley Louis Bridges, John M. Davis

**Affiliations:** ^1^ Division of Rheumatology Mayo Clinic Rochester MN USA; ^2^ Division of Clinical Immunology and Rheumatology University of Alabama at Birmingham Birmingham AL USA

**Keywords:** autoantibody, autoimmune, evolution, gender, immunoglobulin

## Abstract

Women have up to a fourfold increase in risk for autoimmune disease compared to men. Many explanations have been proposed, including sex hormones, the X chromosome, microchimerism, environmental factors, and the microbiome. However, the mechanism for this autoimmune sex bias remains obscure. In this manuscript, we evaluate the hypothesis that qualitative or quantitative differences in circulating antibodies may explain, at least in part, the pathogenesis of autoimmune disease and its sex bias—especially when considering an evolutionary perspective. Indeed, women have higher absolute levels of antibodies than men, and (auto)antibodies are also associated with most autoimmune diseases. Several facts suggest differences in antibodies may cause increased prevalence of autoimmune disease in women. First, the association between increased quantities of serum antibodies and increased prevalence of autoimmunity is found not only in women, but also in men with Klinefelter syndrome. Second, both serum antibody levels and autoimmunity spike in the postpartum period. Third, a dose–response effect exists between parity and both serum antibodies and prevalence of autoimmune disease. Fourth, many biologically plausible mechanisms explain the association, such as T cell‐dependent activation of B cells and/or VGLL3. The evolutionary underpinning of increased antibodies in women is likely to be protection of offspring from infections. Overall, this evolutionary paradigm can help explain why the phenomenon of autoimmunity occurs preferentially in women and raises the possibility of new treatment options.

The question of why women have more autoimmune diseases than men has intrigued patients, clinicians, and researchers for decades. Answering this question is critical, as autoimmune disease is a leading cause of morbidity and mortality in young and middle‐aged women (Walsh & Rau, 2000). Doing so would not only help explain the pathogenesis of autoimmunity, but inform ways to treat it. Although autoimmunity is a highly complex phenomenon, from an evolutionary perspective, the answer to the question of why women have more autoimmune disease than men may be relatively straightforward.

It is clear that for many autoimmune syndromes, women have a much higher burden of autoimmune diseases compared to men. For example, in systemic lupus erythematosus (SLE) and Sjögren's disease, women have more than a fourfold relative risk (Ji et al., 2016). Many explanations have been proposed such as differences in sex hormones, an obvious biological difference between women and men. Researchers have reported that estrogen increases T‐cell calcineurin expression in patients with SLE (Rider et al., 1998), androgens decrease expression of the pathogenic *FcgammaRIIB2* gene in Graves' disease (Estienne et al., 2002), progesterone induces autoimmune thyroiditis in rats (Ansar Ahmed et al., 1983), and prolactin increases SLE activity in mice (McMurray et al., 1991). Although these data support the hypothesis we propose, there has been much conflicting data (Natri et al., 2019; Ngo et al., 2014). Additional hypotheses for the sex bias include abnormal X chromosome inactivation (Ozcelik et al., 2006; Simmonds et al., 2014; Syrett et al., 2019), microchimerism (Nelson, 2012), differences in environmental exposures such as cosmetic chemicals or sunlight (Fraser et al., 2003; Ngo et al., 2014; Ponsonby et al., 2005; Rieger et al., 2006; Shapira et al., 2010), and the microbiome (Gomez et al., 2015; Johnson et al., 2020; Markle et al., 2013; Yurkovetskiy et al., 2013). All of these factors may play a role in sex‐related differences in autoimmunity, but there has been no satisfactory overall paradigm of how these components explain why women are more prone to develop autoimmune diseases (Natri et al., 2019; Ngo et al., 2014).

Although a bit simplified, one underlying factor may more simply explain a significant portion of the autoimmune sex bias: antibodies. Specifically, we hypothesize that women have an evolutionarily conserved tendency toward enhanced B‐cell activation and production of higher levels of antibodies, which results in increased incidence of antibody‐driven autoimmune diseases. This hypothesis stems from our observation that women have more serum immunoglobulins (i.e., antibodies) than men at baseline (Butterworth et al., 1967; Crisp & Quinn, 2009; Dillon et al., 2020; Rowley & Mackay, 1969) and in response to challenges like infection or vaccination (Cook, 2008; Rowley & Mackay, 1969). While this difference may peak in the first two decades of life (Oyeyinka et al., 1984), women seem to have more absolute antibody levels than men lifelong (Crisp & Quinn, 2009; Dillon et al., 2020). A recent study showed that women have not only more antibodies in general, but also autoantibodies (Dillon et al., 2020). This finding is significant, as autoantibodies responsible for many autoimmune diseases, especially the ones which are more heavily female predominant such as SLE, Sjögren's disease and myasthenia gravis (Fairweather et al., 2008). Furthermore, we hypothesize that the increased B‐cell activation and antibody production in women is not as an accident, but rather, an evolutionary advance to protect offspring from infectious diseases. As a general principle, this hypothesis may more simply and accurately and explain part of the sex bias in autoimmune disease and thereby illuminate future efforts to understand and treat these conditions.

Many scientific facts at first seem to contradict this hypothesis. First, many women with high antibody levels never develop autoimmune disease given its low prevalence in the general population (Ji et al., 2016). Similarly, women do not have more B cells than men (Kverneland et al., 2016; Yan et al., 2010). Importantly, however, a comparison of the number of activated B cells in healthy men and women has not been reported. Third, patients with reduced immunoglobulin levels from common variable immune deficiency (CVID) have increased, rather than decreased, prevalence of autoimmune diseases (Gathmann et al., 2014). However, more recent studies have shown that several identifiable genes related to the immune system confound this relationship by causing both the CVID and autoimmune disease, such as loss of function in *NFKB1* and *NFKB2*, *PRKCD*, and *TACI* (Kitcharoensakkul & Cooper, 2019). Fourth, administering antibodies via intravenous immunoglobulin (IVIG) ameliorates rather than worsens many autoimmune diseases (Patil & Kaveri, 2013). Nevertheless, IVIG's mechanism of action involves not only competing with pathogenic antibodies, but also inhibiting the expansion of autoreactive B cells (Patil & Kaveri, 2013), which is consistent with our hypothesis. In addition, some autoimmune diseases such as rheumatoid arthritis (RA) have peak incidence after child‐bearing years (mean age 55) when increased antibodies would not be protective to offspring (Myasoedova et al., 2020). However, autoantibodies are increasingly recognized to occur many years before the onset of clinical disease (Arbuckle et al., 2003; Nielen et al., 2004; Rantapaa‐Dahlqvist et al., 2003). Finally, the association between antibodies and autoimmune disease might simply reflect correlation or an epiphenomenon rather than causation.

Nevertheless, several facts suggest the relationship between antibodies and autoimmune disease might indeed be causal. First, the association between increased immunoglobulins and risk of autoimmune disease is found not just in women, but also in other populations including men with Klinefelter syndrome (Scofield et al., 2008; Seminog et al., 2015) as well as in mice (Jones et al., 2019) and insects (Kelly et al., 2018). Second, a temporal sequence of association seems to occur in the postpartum period, when both antibodies (Jansson et al., 1987) and autoimmune diseases are known to spike (Jorgensen et al., 2012), particularly antibody‐mediated autoimmune diseases like SLE and thyroid disease (Ngo et al., 2014). Third, a dose–response effect exists between the number of children and risk of autoimmune diseases such as RA and autoimmune thyroid disease (Carle et al., 2014; Jorgensen et al., 2012) and between the number of antibodies and risk of type 1 diabetes (Fairweather et al., 2008). Fourth, a recent experimental study showed that administering rituximab (an anti‐B‐cell agent) to individuals with pre‐RA‐delayed disease onset (Gerlag et al., 2019). This finding supports a role of B cells, and/or the antibodies they produce, in RA pathogenesis.

Several potential biological mechanisms explain how the relationship between antibodies and autoimmune disease might occur, especially involving B‐cell activation. First, CD40L‐CD40 ligation is critical for B‐cell proliferation to occur, and CD40L is overexpressed on both T‐cell surface (Koshy et al., 1996) and serum (Kato et al., 1999) in patients with SLE. Furthermore, CD40 has been shown to play a pathogenic role in numerous other autoimmune diseases including type 1 diabetes, inflammatory bowel disease, multiple sclerosis and RA (Peters et al., 2009). Indeed, men may have fewer circulating T cells than women, further supporting a T cell‐driven mechanism for the autoimmune sex bias (Bouman et al., 2004; Kverneland et al., 2016; Ohta et al., 1986; Yan et al., 2010). Recently, studies have also shown that women have skin‐targeted overexpression of *VGLL3,* which encodes a transcription factor that drives pro‐inflammatory genes including B‐cell activating factor (Billi et al., 2019; Liang et al., 2017). This finding was independent of age and sex hormone regulation (Liang et al., 2017). Such enhanced B‐cell activation explains how women might have increased total serum antibodies without increased numbers of B cells. Importantly, other genes associated with autoimmune disease such as *PTPN22* and *STAT4* play roles in both lymphocyte activation and establishment of tolerance, explaining why women might be more prone not only to increased antibodies, but increased *pathogenic* antibodies through errors in the fidelity of self‐tolerance (Bottini & Peterson, 2014; Korman et al., 2008). Finally, sex hormones might also drive the relationship, as estrogen increases antibody production by lymphocytes (Kanda & Tamaki, 1999), while androgens decrease antibody levels (Kocar et al., 2000). Indeed, the imperfect associations between sex hormones, the X chromosome, and the microbiome with autoimmunity may be explained by the fact that these are mediators of the true association between antibodies and autoimmune disease, rather than the cause themselves.

Evolutionarily, why would nature preserve excessive autoantibody production in women, especially when it can be so maladaptive during prime child‐bearing years? The answer seems to be infections. For most of human history, infections were unequivocally the leading cause of death for both adults and infants (Finch, 2010). In fact, despite recent medical advances, infections still remain the leading cause of infant and child mortality worldwide, with 69% of child deaths under 5 attributed to infections (Causes of Child Mortality, 2017). Thus, evolution would have preserved any mechanism to reduce the chance of infections, regardless of the cost.

Antibodies protect both mothers and their offspring (via breastmilk) from infection during the vulnerable infant period. Indeed, women, who are known to have more antibodies, have better outcomes and survival from bacterial, parasitic, and viral infections (Choudhry et al., 2006; Gannon et al., 2004; Natri et al., 2019; Ngo et al., 2014). Furthermore, genes that predispose to autoimmunity are also known to have excellent pathogenic peptide presentation capabilities for clearing infections (Mangalam et al., 2013). Moreover, a recent study showed that sex hormones strongly influence thymic epithelial cell biology, providing a strong mechanistic explanation for both the decreased susceptibility to infections and increased susceptibility to autoimmune disease observed in females (Dumont‐Lagacé et al., 2015). Although autoimmune disease may have harmed mothers, the reduced infection risk likely enhanced their offspring's survival such that propensity for autoimmunity passed on to the next generation in classic Darwinian fashion.

The hypothesis that increased antibodies lead to increased autoimmunity in women could be tested in a number of ways. First, assuming that B‐cell activation is the next proximal step in the causal pathway, researchers could measure activated B cells in healthy women compared to healthy men, as well as women and men with a particular autoimmune disease. Comparing women and men with regard to the number of B cells and plasma cells in lymphoid organs or the bone marrow (where the largest proportion of antibody‐producing plasma cells exist) would also be revealing. To more directly address our hypothesis, a study could also measure total immunoglobulins in unaffected individuals (e.g., from donated blood), and then evaluate risk of later developing autoimmune disease. Prospective trials, such as the ongoing APIPPRA study for pre‐RA (Al‐Laith et al., 2019), should provide information on the effect of blocking B‐cell activation on development of autoimmune disease in high‐risk individuals.

If confirmed, this hypothesis would have several implications for science and medicine. First, it would help scientists, clinicians, and patients to understand why the autoimmune sex bias occurs. For patients in particular who deal with sometimes devastating manifestations of autoimmune disease, this understanding may be comforting. That is, their development of an autoimmune disease is an adaptation that balances protecting their children at the cost of a higher tendency to autoimmunity. Second, it might reveal novel ways to treat autoimmune disease, for example, through inhibiting polyclonal B‐cell activation. Furthermore, such therapies could eventually be used for prevention of autoimmune disease, thus eliminating their often debilitating consequences.

## CONFLICTS OF INTEREST

None.
